# Preinvasive and invasive disease in women with cytological diagnosis of high-grade lesion and high-grade lesion cannot exclude microinvasion

**DOI:** 10.1186/s12905-015-0239-5

**Published:** 2015-10-08

**Authors:** Nina de Siqueira Kuperman, Fábio Bastos Russomano, Yara Lucia Mendes Furtado de Melo, Saint Clair dos Santos Gomes

**Affiliations:** Instituto Nacional de Saúde da Mulher, da Criança e do Adolescente Fernandes Figueira, Fundação Oswaldo Cruz (IFF/Fiocruz), Av. Rui Barbosa, 716 - Flamengo, CEP 22250-020 Rio de Janeiro, Brazil; Department of Gynecology, Colposcopy Clinic, Instituto Nacional de Saúde da Mulher, da Criança e do Adolescente Fernandes Figueira, Fundação Oswaldo Cruz (IFF/Fiocruz), Rio de Janeiro, Brazil; Department of Gynecology, Universidade Federal do Estado do Rio de Janeiro (HUGFF/UNIRIO), Rio de Janeiro, Brazil; Department of Clinical Research, Instituto Nacional de Saúde da Mulher, da Criança e do Adolescente Fernandes Figueira, Fundação Oswaldo Cruz (IFF/Fiocruz), Rio de Janeiro, Brazil

**Keywords:** Uterine cervical neoplasms, Cervical intraepithelial neoplasia, Vaginal smears

## Abstract

**Background:**

Cervical cancer is the third most common cancer in Brazil and has a high potential for prevention and cure. The prevalence of invasive and preinvasive disease in women with cytological diagnosis of high-grade lesion – cannot exclude microinvasion (HSIL-micro) is not known.

**Methods:**

This cross-sectional study used a cytology lab database to identify women with HSIL-micro and HSIL referred to two colposcopic units from June 2006 to December 2012. For each woman with HSIL-micro, four women with cytologic diagnosis of HSIL who met the inclusion criteria were identified. Data were obtained from review of medical records.

**Results:**

Forty-seven patients with report of HSIL-micro and 188 patients with report of HSIL were included. The final diagnoses revealed a frequency of preinvasive lesions of 31.9 % (15/47) and 59.6 % (112/188) in patients with HSIL-micro and HSIL, respectively, while the frequency of invasive disease was 63.8 % (30/47) and 11.7 % (22/188), respectively. The HSIL-micro group showed prevalence of preinvasive or invasive disease 6.5 times greater (95 % CI = 1.6-5.7) and, for invasive disease, 2.4 times greater (95 % CI = 1.7-3.6) than the HSIL group.

**Conclusion:**

Higher risk of preinvasive and invasive lesions in women with cytologic diagnosis of HSIL-micro reinforces recommendations for immediate investigation.

## Background

Cervical cancer is the fourth most common cancer in women, and the seventh overall, with an estimated 528,000 new cases in 2012. Also 266,000 deaths from cervical cancer were estimated worldwide in 2012, accounting for 7.5 % of all female cancer deaths http://globocan.iarc.fr/old/FactSheets/cancers/cervix-new.asp.

The main prevention strategy is based on screening programs using the Pap smear and reference for colposcopy in positive cases, according to specific guidelines. Almost 85 % of cancer cases occur in developing countries, where screening is less effective. The highest incidence rates are observed in Latin America and in the Caribbean, in sub-Saharan Africa, as well as in south and southeast Asia http://globocan.iarc.fr/old/FactSheets/cancers/cervix-new.asp [1].

Cervical cancer is the third most common cancer among women in Brazil. In 2014, 15,590 new cases of cervical cancer were expected, with an estimated risk of 15.33 cases per 100,000 women [2].

Screening of cervical cancer and its precursors in Brazil is performed by a cervical Pap smear, every 3 years in 25–64 year-old women, in an opportunistic manner [2]. The nomenclature for cytologic reports [3] is based on the Bethesda Classification, which is the most accepted and worldwide used nomenclature for Pap smear reports. However, in Brazil, a diagnostic category not found in the original Bethesda System, the “High-Grade Squamous Intraepithelial Lesion cannot exclude microinvasion” (HSIL-micro) is also possible. This category can be considered analogous to the situation foreseen in the Bethesda System, in which it is possible to register the observation "microinvasion cannot be excluded" within the High-Grade Squamous Intraepithelial Lesion category (HSIL) [4].

The Brazilian Guidelines for Cervical Cancer Screening aim to standardize recommendations for proper care of women identified as possible carriers of preinvasive or invasive lesions.

For patients with a Pap smear indicating HSIL-micro, the recommendation is immediate referral to a secondary care unit for colposcopy. When colposcopy shows no lesion or the findings are not suggestive of invasion, the recommended approach is excision of the transformation zone (ETZ), according to its location [5]. If changes suggestive of invasion are present, one or more biopsies including representative specimens of the lesion should be submitted to further analysis.

This approach is more invasive than that recommended to women with HSIL report, in which some investigative procedures are necessary before an excisional procedure, when major findings are not seen.

However, the recommendation for women with diagnosis of HSIL-micro is based on expert opinion, since there is no information on the risk of preinvasive or invasive lesions in women with this cytological diagnosis.

The purpose of this study is to estimate the risk, through prevalence ratio (PR), of preinvasive and invasive lesions in women with HSIL-micro Pap smear reports compared with those with HSIL. In other words, since patients with a cytologic result of HSIL-micro must be submitted to ETZ, our aim is to find out whether the histological results of these patients significantly differ from those of patients with HSIL cytology in order to support this more invasive approach.

## Methods

### Study design

A cross-sectional study with women identified in the SITEC database (National Cancer Institute Technological Integrated Service in Cytology), which examines all Pap smear samples from women visiting primary care units in the city of Rio de Janeiro (RJ, Brazil). Data were obtained from medical records.

### Subjects

The study located all women with HSIL-micro report in cytologic exams obtained between June 2006 and December 2012 and who were referred and assisted in one of the collaborating secondary units: Instituto Nacional de Saúde da Mulher, da Criança e do Adolescente Fernandes Figueira, Fundação Oswaldo Cruz (IFF/Fiocruz), Rio de Janeiro, Brazil (IFF/Fiocruz, RJ, Brazil) and Instituto Nacional do Câncer José Alencar Gomes da Silva (Inca, RJ, Brazil). Pregnant women and patients with some type of immunodeficiency were excluded. For each patient included with HSIL-micro diagnosis, four patients with HSIL cytologic diagnosis referred and assisted in one of these units were included: two diagnosed immediately before and two immediately after each diagnosis of HSIL-micro.

### Variables and outcomes

Information related to the investigation and final diagnoses were obtained from medical records of the included patients. We searched for patient characteristics (age, number of gestations, number of child births) colposcopic findings, performed procedures and final histological or follow-up diagnosis.

### Statistical analysis

Descriptive analyses were performed to estimate frequencies, profiles and identify outliers. Bivariate analyses were performed to estimate association between Pap smear and final diagnosis. Excel® was used to create the database and SPSS® to perform the statistical analysis. Student’s t-test was used to analyze numerical variables. Chi-square test or Fisher's exact test were used for categorical variables. All analyses were performed considering the confidence level of 95 %.

### Ethical issues

This study was approved and informed consent waived by the ethics committees of the IFF/Fiocruz and Inca under protocol numbers 213.239 and 132/12, respectively.

## Results

Between June 2006 and December 2012, 96,855 Pap smears were analyzed by SITEC. Among these, 4,581 were referred to Inca and IFF/Fiocruz, the colposcopic units participating in our study.

**Fig. 1:**
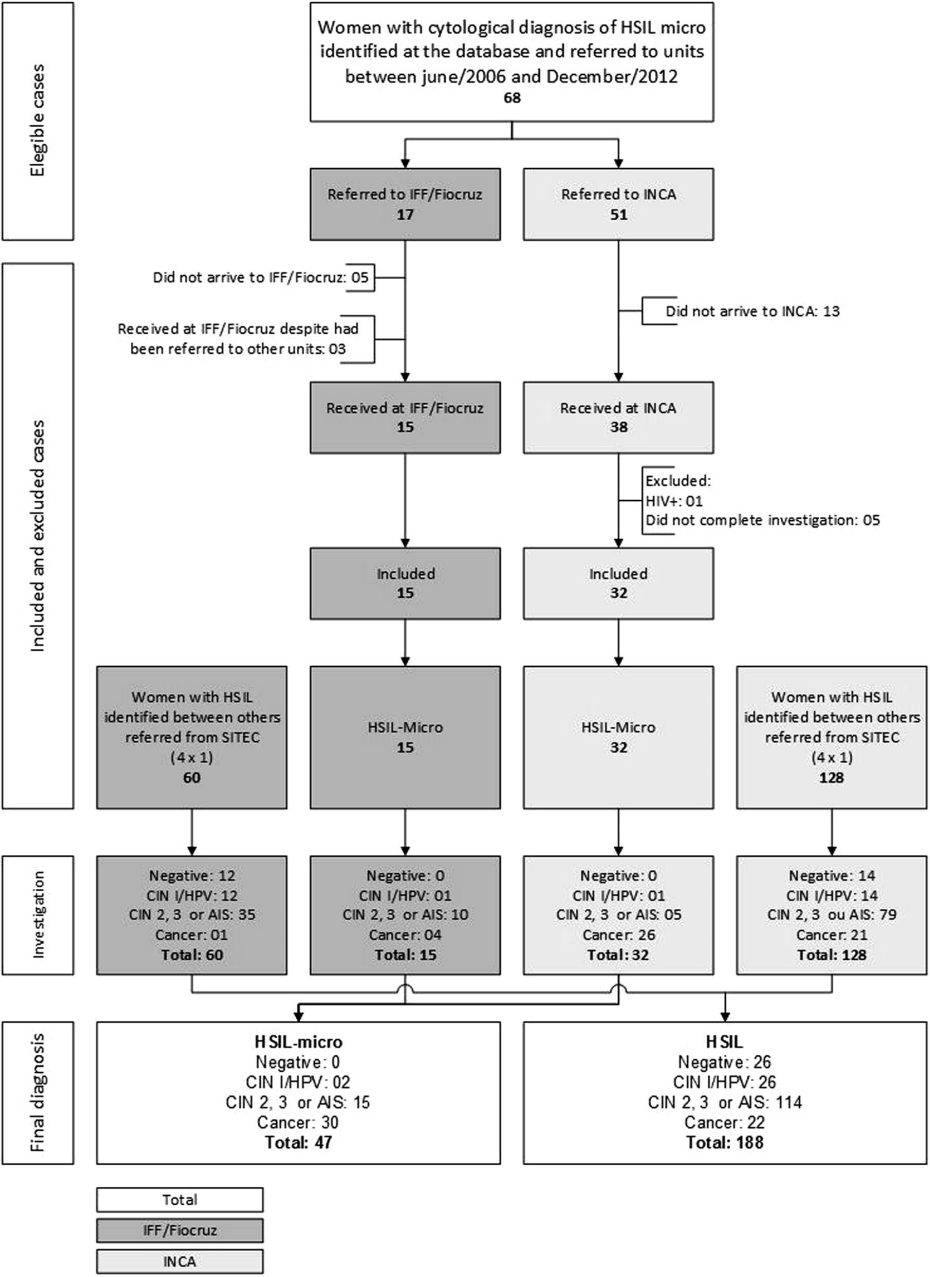
Flowchart of the study

We identified 318 patients with HSIL-micro cytologic diagnosis, of whom 68 were referred to the following collaborating units: 17 to IFF/Fiocruz and 51 to Inca in this period of time (Fig. 1). Out of these, 15 and 38 patients, respectively, were actually received at the units. The 15 patients received at IFF/Fiocruz were included in the study. Of the 38 patients received at Inca, six were excluded (one tested positive to Human Immunodeficiency Virus - HIV - and five did not conclude the diagnostic investigation). Therefore, thirty-two women were included in the study. To compose the comparison group, 60 cases of HSIL were included at IFF/Fiocruz and 128 at Inca (a 4:1 HSIL:HSIL-micro ratio, to increase study power).

The sample characteristics are described in Table 1. The mean age was 49.78 years for patients with HSIL-micro cytologic report and 34.36 years for patients with a HSIL report (*p* <0.0001). There were also differences in parity: in the first group, patients had an average of 4.56 pregnancies and 3.82 deliveries compared to an average of 2.68 pregnancies and 2.27 deliveries in the second group (*p* = 0.018 and 0.006, respectively).

**Table 1:** Sample characteristics (IFF/Fiocruz-INCA, RJ, Brazil, 2006–2012)

Characteristic	HSIL micro	HSIL	Total	*p*-value
*N* (%)	47 (20)	188 (80)	235 (100)	-
Age (mean, SD)	49.78 (14.44)	34.36 (11.84)	37.44 (13.83)	<0.001^a^
Number of pregnancies	4.56 (4.05)	2.68 (1.94)	3.14 (2.71)	<0.018^a^
Parity	3.82 (3.53)	2.27 (1.76)	2.60 (2.34)	<0.006^a^
Follow-up unit *n* (%)				
IFF	15 (31.9)	60 (31.9)	75 (31.9)	0.999
INCA	32 (68.1)	128 (68.1)	160 (68.1)	
Colposcopic findings *n* (%)				
Normal	3 (6.4)	12 (6.4)	15 (6.4)	
Minor abnormal findings (grade 1)	2 (4.3)	25 (13.3)	27 (11.5)	
Major abnormal findings (grade 2)	15 (31.9)	67 (35.6)	82 (34.9)	<0.001
Suspicious for invasion	6 (12.8)	0 (0)	6 (2.6)	
Not reported	21 (44.7)	84 (44.7)	105 (44.7)	
View of SCJ^b^ *n* (%)				
Visible SCJ	6 (12.8)	61 (32.4)	33 (14)	
Non visible or partially visible SCJ	14 (29.8)	19 (10.1)	67 (28.5)	<0.001^c^
Not reported	27 (57.4)	108 (57.4)	135 (57.4)	
Procedure *n* (%)				
None	0 (0)	16 (8.5)	16 (6.8)	
Biopsy	19 (40.4)	43 (22.9)	62 (26.4)	
Type 1 excision^d^	7 (14.8)	102 (54.3)	109 (46.4)	<0.001
Type 3 excision^e^	15 (31.9)	22 (11.7)	37 (15.7)	
Not reported	5 (10.6)	5 (2.6)	10 (4.3)	

^a^Student's t-Test

^b^Squamous Columnar Junction

^c^Chi-square test (excluded cases with no data)

^d, e^Using Large Loop or Straight Wire Excision of the Transformation Zone

In the HSIL-micro group, 31.9 % of patients had major findings and 12.8 % had findings suggestive of invasion on colposcopic examination. In the HSIL group, major findings were found in 35.6 % and no cases had findings suggestive of invasion. There was a significant difference between groups regarding visualization of the squamous columnar junction (SCJ). In 29.8 % of patients with cytological report of HSIL-micro the SCJ was not visible, while 10.1 % of patients with cytological report of HSIL had a non-visible SCJ (Table 1).

The final diagnoses revealed a frequency of preinvasive lesions (Cervical Invasive Neoplasia - CIN - 2–3 or adenocarcinoma *in situ* - AIS) of 31.9 % (15/47) and 59.6 % (112/188) in patients with HSIL-micro and HSIL, respectively, while the frequency of invasive disease (IA1 cancer, invasive cancer and adenocarcinoma) was 63.8 % (30/47) and 11.7 % (22/188), respectively (Table 2).

**Table 2:** Final diagnoses obtained from each group (IFF/Fiocruz-INCA, RJ, Brazil, 2006–2012)

	Pap smear diagnosis *n* (%)		Total	
Final diagnosis^a^	HSIL micro^h^	HSIL^c^		*p*-value
Negative	0 (0)	24 (12.8)	24 (10.2)	0.005
LSIL^b^	2 (4.3)	28 (14.9)	30 (12.8)	0.052
HSIL^c^	15 (31.9)	112 (59.6)	127 (54)	<0.001
AIS^d^	0 (0)	2 (1.1)	2 (0.9)	1.000
Microinvasive lesion^e^	6 (12.8)	10 (5.3)	16 (6.8)	0.070
Invasive lesion^f^	19 (40.4)	9 (4.8)	28 (11.9)	<0.001
Adenocarcinoma^g^	5 (10.6)	3 (1.6)	8 (3.4)	0.009
Total (%)	47 (100)	188 (100)	235 (100)	

^a^Diagnoses obtained at the end of investigation considering histopathological specimens. Negative cases were based on absence of visible colposcopic findings and two negative cytopathologic exams with a minimum of six-month intervals

^b^Low-grade squamous intraepithelial lesion (includes cytopathic effect by HPV and CIN 1)

^c^High-grade squamous lesion (includes CIN 2 and CIN 3)

^d^Adenocarcinoma *in situ*

^e^Microinvasive squamous carcinoma of the cervix

^f^Invasive squamous carcinoma of the cervix

^g^Invasive squamous adenocarcinoma of the cervix

^h^High-grade intraepithelial lesion cannot exclude microinvasion

Table 3 shows the distribution of the final diagnoses recategorized as cancer or preinvasive lesions (CIN 2–3 or AIS) and the absence of these diagnoses (CIN 1, Human Papilloma Virus - HPV - cytopathic effect and negative results) by cytological diagnosis (HSIL-micro versus HSIL). In the HSIL-micro group, 95.7 % (45) had either preinvasive or invasive disease, while in the HSIL group, 72.3 % (136) had one of these lesions. There was a significant difference between groups (*p* < 0.0001), the HSIL-micro group had a prevalence of preinvasive or invasive disease 6.5 times (95 % CI = 1.6-5.7) higher than the HSIL group.

**Table 3:** Prevalence of preinvasive or invasive disease by study group (IFF/Fiocruz-INCA, RJ, Brazil, 2006–2012)

Final diagnosis^a^	Preinvasive or invasive lesion^b^	Absence of preinvasive or invasive lesion^c^	Total *n* (%)	Prevalence Ratio (PR) (CI 95 %)
Pap smear diagnosis				
HSIL-Micro^d^	45	2	47	6.5 (1.6 - 25.7)
HSIL^e^	136	52	188	<0.001
Total	181	54	235	

^a^Recategorized diagnoses obtained at the end of investigation considering histopathological specimens. Negative cases were based on absence of visible colposcopic findings and two negative cytopathologic exams with a minimum of six-month intervals

^b^Includes CIN 2, CIN 3, adenocarcinoma in situ and microinvasive or invasive squamous carcinoma of the cervix

^c^Includes negative diagnoses, HPV cytopathic effect and CIN 1

^d^High-grade intraepithelial lesion cannot exclude microinvasion

^e^High-grade intraepithelial lesion

Table 4 shows a similar analysis, but correlating the cytologic diagnosis with presence or absence of invasive lesion in comparison with non-invasive lesions or no lesions at all. In the HSIL-micro group, 63.8 % (30) had cancer as a final diagnosis. In the HSIL group, 11.7 % (22) had a final diagnosis of cancer. There was also a significant difference between groups (*p* < 0.0001), the HSIL-micro group showed prevalence of invasive disease 2.4 times (95 % CI = 1.7-3.6) higher than HSIL group.

**Table 4:** Prevalence of invasive disease by study group (IFF/Fiocruz-INCA, RJ, Brazil, 2006–2012)

Final diagnosis^a^	Invasive lesion^b^	Absence of invasive lesion^c^	Total *n* (%)	Prevalence Ratio (PR) (CI 95 %)
Pap smear diagnosis				
HSIL-micro^d^	30	17	47	2.4 (1.7 – 3.6)
HSIL^e^	22	166	188	<0.001
Total	52	183	235	-

^a^Recategorized diagnoses obtained at the end of investigation considering histopathological specimens. Negative cases were based on absence of visible colposcopic findings and two negative cytopathologic exams with a minimum of six-month intervals

^b^Includes microinvasive squamous carcinoma of the cervix

^c^Includes negatives, CIN 1, 2 or 3, or AIS

^d^High-grade intraepithelial lesion cannot exclude microinvasion

^e^High-grade intraepithelial lesion

## Discussion

The search for information published at PubMed, Cielo, Embase and Lilacs databases, concerning the prevalence of preinvasive or invasive disease in the presence of HSIL-micro cytological diagnosis, proved to be unsuccessful at a national and international level. The terms used in these searches were “Cervical Intraepithelial Neoplasia” and “Papanicolaou Test” and either one of the following: “HSIL microinvasion”, “microinvasion”, “cannot exclude microinvasion”, “cannot exclude micro invasion” or “cannot exclude invasion”. As no scientific article was found on this cytological diagnosis, it was not possible to make a direct comparison between our results and those from other authors.

In this study, women presenting HSIL in Pap smear results had preinvasive lesions in 60.7 % of the cases. Adding such results to the invasive lesion cases, the percentage of both lesions reached up to 72.3 %. This percentage is similar to that described in the medical literature, which is about 70 % [6].

Still, Massad et al. [7] studied the correlation between cytologic and histologic diagnoses, and found in women with HSIL cytologic results, 47.8 % of CIN 2 or 3 and 5 % of cancer. This lower percentage may be explained by the difference in women’s age, since the mean age of women included in their study was lower than that found in our study (33 *vs*. 37.4 years old) [7].

The frequency of invasive disease in women with HSIL in our study was 11.7 % (22/188), which is much higher than the numbers reported by Massad et al. [7], who found it in 5 % of their sample. Other studies mention higher frequencies of CIN2-3 in women with a Pap smear report of HSIL [8–11]. These frequencies depend upon the prevalence of preinvasive disease in the population and may explain the discrepancies observed in different studies.

Our higher frequency of invasive and pre invasive disease can be explained by the fact that the screening program in Brazil is opportunistic. Although the screening is offered to all women between 25 (if sexually active) and 64 years old, controlling underscreened women is impossible due to the current lack of a population-based information system [2].

However, in women with HSIL-micro cytologic reports, preinvasive and invasive diseases were present in 31.9 % and 63.8 % of them, respectively. These findings show that nearly all women with this cytologic diagnosis (95.7 %) have a significant disease and require investigation and treatment, reinforcing the recommendation of excisional procedures in such cases. Although it is not possible to make a direct comparison of these values with data from other publications, we consider these findings quite reliable and generalizable to a similar sample.

The prevalence ratio of invasive disease and preinvasive plus invasive lesions in the HSIL-micro group compared with the HSIL group was 2.41 and 6.5, respectively, showing that the former actually points to a greater risk of significant disease.

Although the number of pregnancies, parity, visibility of SCJ and performed procedure were statistically related to the cytological report, these factors are interrelated and age-related, as older women tend to have more children, their SCJ tend to be endocervical and, as a consequence, more type 3 excisions are expected to be performed. Similarly, colposcopic findings are *proxies* of the outcome itself and cannot act as a confounder (Table 1).

The referral unit, another possible confounder due to differences in routine care was not significantly related to the cytological report (Table 1).

We analyzed age as a possible confounder, but the differences between age groups were not significant, due to the small number of patients in some age ranges (data not shown).

## Conclusion

The higher frequency of preinvasive and invasive lesions in women with cytologic diagnosis of HSIL-micro reinforces recommendations for immediate investigation.

## Abbreviations

HSIL-micro: High-grade squamous intraepithelial lesion cannot exclude microinvasion
PR: Prevalence ratio
HSIL: High-grade squamous intraepithelial lesion
CI: Confidence interval
ETZ: Excision of transformation zone
SITEC: Seção Integrada de Tecnologia em Citopatologia of Instituto Nacional de Câncer
IFF/Fiocruz: Instituto Nacional de Saúde da Mulher, da Criança e do Adolescente Fernandes Figueira of Fundação Oswaldo Cruz
Inca: Instituto Nacional de Câncer José Alencar Gomes da Silva
HIV: Human immunodeficiency virus
SCJ: Squamous columnar junction
CIN: Cervical intraepithelial neoplasia
HPV: Human papilloma virus
